# Mortality Predictors and Neurological Outcomes Following Extracorporeal Cardiopulmonary Resuscitation (eCPR): A Single-Center Retrospective Study

**DOI:** 10.3390/jcdd11090272

**Published:** 2024-09-02

**Authors:** Sasa Rajsic, Helmuth Tauber, Robert Breitkopf, Corinna Velik Salchner, Fabian Mayer, Ulvi Cenk Oezpeker, Benedikt Treml

**Affiliations:** 1 Department of Anaesthesiology and Intensive Care Medicine, Medical University Innsbruck, Christoph-Probst-Platz 1, 6020 Innsbruck, Austria; 2 Department of Cardiac Surgery, Medical University Innsbruck, 6020 Innsbruck, Austria

**Keywords:** cardiopulmonary reanimation, resuscitation, CPR, eCPR, ECMO, outcome, mortality, adverse events

## Abstract

Background: Extracorporeal cardiopulmonary resuscitation (eCPR) offers cardiorespiratory support to patients experiencing cardiac arrest. However, this technology is not yet considered a standard treatment, and the evidence on eCPR criteria and its association with survival and good neurological outcomes remains scarce. Therefore, we aimed to investigate the overall mortality and risk factors for mortality. Moreover, we provide a comparison of demographic, clinical, and laboratory characteristics of patients, including neurological outcomes and adverse events during support. Methods: This retrospective analysis included in-hospital and out-of-hospital cardiac arrest patients who received eCPR and were admitted between January 2008 and June 2022 at a tertiary and trauma one-level university hospital in Austria. Results: In total, 90 patients fulfilled inclusion criteria, 41 (46%) patients survived until intensive care unit discharge, and 39 (43%) survived until hospital discharge. The most common cause of cardiac arrest was myocardial infarction (42, 47%), and non-shockable initial rhythm was reported in 50 patients (56%). Of 33 survivors with documented outcomes, 30 had a good recovery as measured with Cerebral Performance Category score, 2 suffered severe disability, and 1 remained in a persistent vegetative state. Finally, multivariate analysis identified asystole as initial rhythm (HR 2.88, *p* = 0.049), prolonged CPR (HR 1.02, *p* = 0.043), and CPR on the weekend (HR 2.57, *p* = 0.032) as factors with a higher risk of mortality. Conclusions: eCPR-related decision-making could be additionally supported by the comprehension of the reported risk factors for mortality and severe disability. Further studies are needed to elucidate the impact of peri-arrest variables on outcomes, aiming to improve patient selection.

## 1. Introduction

Extracorporeal cardiopulmonary resuscitation (eCPR) is an advanced life support technique employing extracorporeal membrane oxygenation (ECMO) for cardiorespiratory support in patients with cardiac arrest, particularly when conventional cardiopulmonary resuscitation (CPR) is failing [1,2,3,4,5]. By bridging the time needed for the diagnosis and treatment of potentially reversible cardiac arrest causes, it provides oxygenated blood to vital end-organs, augmenting perfusion during the critical phase [1].

The latest International Liaison Committee on Resuscitation (ILCOR) and European Resuscitation Council (ERC) guidelines recommend consideration of eCPR as a rescue therapy for cardiac arrest patients when conventional advanced life support measures are failing or to enable specific interventions, such as coronary angiography and percutaneous coronary intervention, pulmonary thrombectomy, rewarming after hypothermia, etc. [6,7]. However, the guidelines do not further define patient selection criteria, reflecting an ongoing need to identify which patients may benefit the most from this invasive technology.

Although the existing literature indicates potential advantages of eCPR over conventional resuscitation, particularly in selected patient cohorts, the level of evidence remains low. Survival rates vary depending on multiple factors, including patient characteristics, the underlying cause, the timing of possible interventions, and expertise. Recent meta-analyses have highlighted a decrease in in-hospital mortality, better neurological outcomes, and improved 30-day survival compared with the conventional approach [8,9,10,11]. However, eCPR is not yet considered a standard treatment and is primarily used in specialised centres with the necessary resources, expertise, and infrastructure.

The evidence on the eCPR initiation criteria and their association with survival and good neurological outcomes remains scarce. This study reviews in-hospital and out-of-hospital cardiac arrest (IHCA and OHCA) eCPR patients from our centre to assess the mortality and the impact of peri-arrest variables on outcomes.

## 2. Materials and Methods

### 2.1. Study Population

We retrospectively reviewed the medical charts of all patients receiving ECMO who were admitted to the intensive care units of the Department of Anaesthesiology and Critical Care Medicine at the Medical University of Innsbruck, Austria. The observation period was from January 2008 to June 2022. All consecutive OHCA and IHCA patients undergoing eCPR were enrolled. Patients who had ECMO initiated shortly after the return of spontaneous circulation (ROSC) and those with a significant amount of missing data in their medical records were excluded.

### 2.2. eCPR Programme and Management

The eCPR programme is perpetually available at our centre. The decision to initiate eCPR is made by the joint judgement of a resuscitation team, including a cardiac surgeon, a cardiac anaesthesiologist, and an intensivist on call. Manual compressions were typically substituted using a mechanical CPR device (Lund University Cardiopulmonary Assist System—LUCAS). In the majority of cases, cannulation was established through femoral access using the Seldinger technique. Anticoagulation was primarily conducted with unfractionated heparin following the Extracorporeal Life Support Organization (ELSO) Anticoagulation Guidelines [12].

Patients were supported with ECMO until they could be weaned off, underwent mechanical circulatory support device implantation, or received a heart transplant. In cases of futility (due to severe brain damage, irreversible heart damage, or multiple organ failure), ECMO support was terminated. In certain cases, organ procurement was performed.

After successful recovery from the underlying pathology and organ dysfunction, weaning from support was initiated by a stepwise reduction in extracorporeal blood flow under close monitoring of the patient’s haemodynamic and echocardiographic measurements. On the basis of clinical assessment and with extracorporeal life support of less than 30% of the total, a trial off was initiated. Discontinuation and decannulation were conducted according to ELSO guidelines [1].

### 2.3. Data Acquisition

We collected sociodemographic patient data; data on disease severity prior to eCPR initiation, underlying diseases, and most likely cause of cardiac arrest; peri-arrest information (primary rhythms, witnessed event, CPR duration, and blood gas analyses); ECMO support duration; adverse events, Glasgow Coma Scale (GCS) score at discharge, and date and cause of death; blood gas and laboratory parameters; and, finally, data on mortality.

Laboratory data were recorded throughout the entire peri-arrest period, starting shortly before eCPR initiation and daily during the support period. The observation period for laboratory parameters was limited to a maximum of 14 days.

Two authors independently checked medical charts and extracted the data into a predesigned case report form.

### 2.4. Outcomes

The primary endpoint of the study was overall ICU mortality with risk factors and predictors of mortality. Secondary endpoints included the comparison of demographic, clinical, and laboratory characteristics of survivors and non-survivors, neurological outcomes, as well as the incidence and type of adverse events during support.

Reported adverse events included haemorrhage, thromboembolic events, insufficient distal leg perfusion, and leg compartment syndrome. Haemorrhagic complications were observed only during ECMO support for a maximum of 14 days. Any bleeding events occurring thereafter were considered unrelated to the ECMO. We used the ELSO definition for bleeding events, defining a major haemorrhage as clinically overt bleeding associated with a haemoglobin decrease of at least 2 g/dL or administration of two or more blood concentrates over 24 h. Pulmonary, retroperitoneal, or central nervous system bleeding and bleeding requiring surgical intervention were further considered major events. Any other noticeable bleeding was considered minor [13].

The location and type of thromboembolic events were gathered from medical records and radiological reports covering the ECMO support period and up to 14 days after support termination. The cause of death was collected from medical documentation, including post-mortem examination reports.

The level of conciseness during the recovery phase was assessed using the GCS at hospital discharge. Functional outcome was measured using the Cerebral Performance Category (CPC) score, which categorises patient outcomes into five categories (death, permanent vegetative state, severe disability, moderate disability, and good recovery) [14,15].

This retrospective study was approved by the Ethics Committee of the Medical University of Innsbruck, Austria (ethics committee number: 1274/2019).

### 2.5. Statistical Analyses

Statistical analyses were performed using SPSS (version 22.0, released 2013, IBM Corp, Armonk, NY, USA). All statistical tests were two-sided with a significance level of 0.05. Depending on the data type and the normality of the distribution, the results are presented as the mean with standard deviation (SD), median (range, minimum–maximum), or frequency (per cent). Independent samples *t*-tests and Mann–Whitney U tests were used. Chi-square and Fisher’s exact tests were used to test differences between nominal data (frequencies). In the univariate Cox regression analyses, the effect of each potential risk factor on mortality was estimated. The significance level for the multivariate model was set to 0.05. To estimate the survival function depending on the initial rhythm (shockable or non-shockable), the log-rank test was used.

## 3. Results

### 3.1. Patient and ECMO Characteristics

During the study period, 116 patients were supported with ECMO for cardiac arrest, and 90 patients received support during cardiopulmonary resuscitation. Twenty-six patients were excluded from the study due to ECMO initiation shortly after ROSC was achieved. The mean age was 53 ± 17 years, with 59 patients being male (66%), as shown in Table 1. Extracorporeal CPR was more frequently initiated in cases of in-hospital cardiac arrest (64% vs. 29%), and myocardial infarction was the most common aetiology (47%). Overall, 41 patients (46%) survived until ICU discharge, and 39 (43%) survived until hospital discharge.

Cardiac arrest was witnessed in 75 patients (83%), with a shockable initial rhythm in 40 patients (44%), as shown in Table 2. In the majority of cases, cardiac arrest occurred on weekdays, with significantly higher survival rates compared with weekends (54% vs. 24% survivors on weekends, *p* = 0.017). The average duration of CPR was 45 (4–220) minutes, significantly longer in non-survivors (*p* = 0.004).

Assessing blood gas parameters shortly before ECMO initiation and during ECMO support, we found significantly lower initial arterial pH (*p* = 0.022) and higher lactate levels (*p* = 0.004) in non-survivors (Table 2). No significant differences were found in the initial venous pH, potassium, or glucose concentration between the compared groups. Measurements of arterial pH, lactate levels, and glucose taken directly and 30 min following ECMO initiation were comparable to those obtained before initiation.

**Figure 1:**
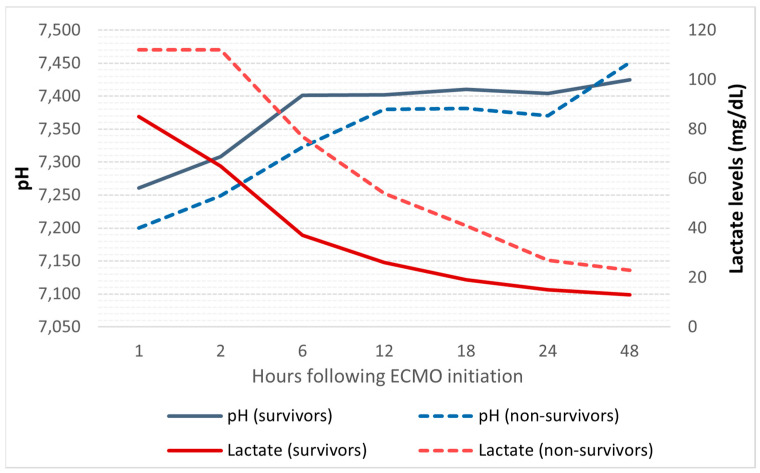
Serial measurements of arterial pH and lactate levels. Abbreviations: ECMO, extracorporeal membrane oxygenation.

Among the blood gas parameters following ECMO initiation, arterial pH was significantly lower in non-survivors during the first six hours, whereas lactate levels remained elevated throughout all observations (Figure 1 and Table S2).

Extracorporeal membrane oxygenation was terminated due to successful weaning in 42 patients (47%), fatality in 37 patients (41%), and bridge to another assist device or heart transplantation in 10 patients (11%), as shown in Table 3.

The most frequent adverse events were acute kidney injury (58%) and haemorrhage (51%), with 47% of patients requiring continuous renal replacement therapy. Major haemorrhage was significantly more common in non-survivors (*p* = 0.004). Death by neurologic criteria was diagnosed in 45% of patients (22), and multiple organ dysfunction syndrome in 35% (17). Additionally, nine patients (20%) underwent organ procurement after being diagnosed with neurological or circulatory death.

### 3.2. Mortality

**Figure 2:**
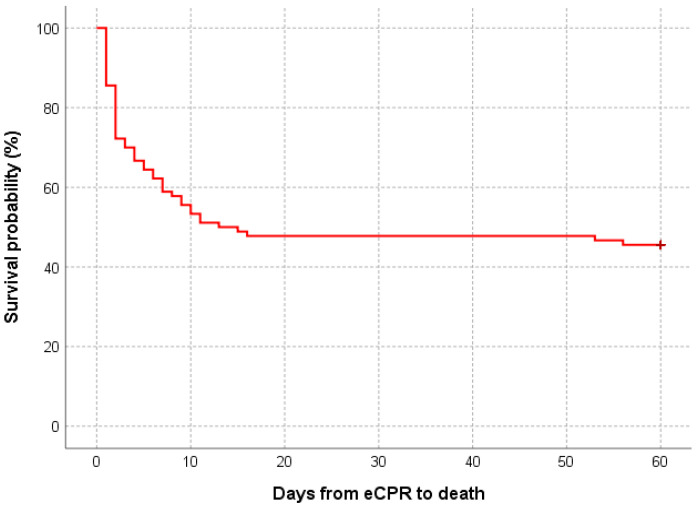
Kaplan–Meier mean estimate of overall ICU mortality. Abbreviations: eCPR, extracorporeal cardiopulmonary reanimation.

Overall ICU mortality was 54% (49 patients, Table 1), and 41% (37) of patients died during ECMO support. Mortality was the highest in case of the cardiac arrest due to pulmonary embolism and hypothermia (caused by water immersion, avalanche, and accidental exposure to a cold environment). The Kaplan–Meier mean estimate of overall ICU mortality was 31 days (n = 90; 95% CI 25.1–36.6, Figure 2). Stratified by initial rhythm, the mean estimate of ICU mortality differed significantly (*p* = 0.044): 25 days for non-shockable (n = 50; 95% CI 17.9–32.9) and 38 days for shockable rhythm (n = 40; 95% CI 29.4–46.2, Supplementary Figure S1).

The univariate Cox regression analysis identified several factors as independent predictors of ICU mortality, including asystole as the initial rhythm, witnessed cardiac arrest, longer duration of CPR, CPR on weekends, hypothermia, OHCA, presence of major haemorrhage, lower arterial pH, and higher lactate before ECMO initiation (Supplementary Table S3). Furthermore, the multivariate Cox regression model revealed that asystole as the initial rhythm (HR 2.88, 95% CI 1.01–8.25), prolonged CPR (HR 1.02, 95% CI 1.00–1.03), and CPR on the weekend (HR 2.57, 95% CI 1.08–6.08) were associated with increased hazard ratios for mortality (Table 4).

### 3.3. Neurological Outcomes

The majority of survivors experienced a favourable recovery following the cardiac arrest, achieving maximum GCS and CPC scores. Seven patients were excluded from the outcome analysis (neurological outcome as measured by CPC) because they were repatriated to home hospitals with a GCS score of three (six patients were sedated and ventilated after ECMO termination, and one patient was on ECMO), making CPC follow-up unfeasible. Of the patients with successful follow-up, 30 survivors achieved a favourable recovery as measured by the CPC (scores 1–2). Two patients experienced severe disability, and one patient remained in a persistent vegetative state.

## 4. Discussion

In this retrospective study conducted at a European university centre, we evaluated the outcomes of patients following eCPR and the impact of peri-arrest variables on ICU mortality. Among the 90 patients included, 46% survived until ICU discharge and 43% were discharged from the hospital, with the majority having favourable neurological outcomes. Multivariate Cox regression analysis identified asystole as an initial rhythm, longer duration of CPR, and CPR on the weekend as factors associated with an increased hazard ratio for ICU mortality. This study presents encouraging findings for both survival and favourable neurological outcomes with ECMO use in patients who failed conventional resuscitation, reaffirming the role of extracorporeal life support in refractory cardiac arrest [4,16,17,18,19,20].

The observed survival-to-hospital discharge rate (43%) is in the upper range of available reports on OHCA (15–33%) and IHCA (30–41%), contributing to the growing evidence on patient outcomes following eCPR [4,21,22]. Given that eCPR may be a promising yet resource-intensive intervention, it is essential to carefully choose patients who are most likely to benefit in terms of achieving good neurological recovery and making efficient use of critical care resources [9,23].

The evidence regarding the impact of the initial rhythm on eCPR patient outcomes is inconclusive. Our findings confirm the association of asystole with higher mortality; patients with asystole had almost a three times higher chance of death compared with others. Daou et al. reported a relationship between shockable rhythm and favourable neurological outcomes in patients resuscitated with eCPR [24]. Similarly, Havranek et al. found that an initial shockable rhythm and eCPR were both associated with neurologically favourable survival [25]. Li et al. identified the presence of pulseless electrical activity or asystole as a mortality risk factor [26]. However, other reports did not find an association between initial rhythm and mortality [21,27,28]. As the use of eCPR is still seen as an emerging technology for this indication, further studies are needed to enhance the available evidence on the role of initial rhythm on patient outcomes, possibly improving patient selection.

On the other hand, prolonged CPR is a well-known predictor of worse outcomes [29,30,31,32,33]. The optimal time interval for eCPR initiation ranges from 30 min to 1 hour after cardiac arrest, with possible extensions beyond this time for selected patients [34,35,36]. The lower threshold is determined by the possibility of achieving ROSC through conventional CPR in the early stage, thus preventing the risk of complications associated with ECMO support [37]. Although ECMO support can be lifesaving in many situations, it may be associated with complications that can lead to permanent injury or death. The upper threshold is not clearly defined, as there are reports on favourable neurological outcomes following prolonged CPR, as demonstrated by numerous case reports and special circumstances [38,39]. Given that the decision to initiate eCPR is often time-critical and based on incomplete information, considering a bridge-to-decision approach may help reduce unnecessary prolongation of low-flow CPR [6,11,37,40]. Therefore, an individualised multidisciplinary decision-making approach may be the best strategy.

Patients with cardiac arrest on weekends had higher mortality. This “weekend effect” is well described for surgical and nonsurgical patients and most likely has a heterogeneous basis [41,42]. In our work, these patients had a higher SAPS III score and suffered more often from hypothermia or myocardial infarction. The higher SAPS III score could be explained by the fact that emergency procedures increase the SAPS III score by five points, and on the weekend, emergencies may dominate over routine work.

Given the above, the initiation of eCPR carries a significant risk of complications, including life-threatening haemorrhage, thromboembolism, acute kidney injury, sepsis, and other adverse events that can impact the quality of life or even cause death [3,43,44]. Haemorrhage is one of the most frequent adverse events during ECMO [3,45,46,47,48,49,50]. In our patient cohort, 51% of patients experienced haemorrhage, and major haemorrhagic events were associated with an increased risk of mortality. These findings are comparable to recent meta-analyses investigating the rate of complications during venoarterial ECMO [3,51,52].

Interestingly, we did not observe a significant association between age and mortality in our cohort of eCPR patients, with the oldest patient being 87 years old (experiencing intraoperative cardiac arrest during transcatheter aortic valve implantation) and the oldest survivor being 74 years old. We initiated ECMO support in 15 eCPR patients older than 70 years, with 47% (7 out of 15) surviving until hospital discharge. All of these patients had good neurological outcomes (data available for five out of seven patients). Earlier reports have shown inconsistent findings, concluding that a rigid interpretation of age as a cut-off for eCPR should be avoided [8,53,54,55,56]. A recent study from the USA reported that age is an independent predictor of neurologically favourable survival to hospital discharge; however, patients between 70 and 79 years of age still had a good outcome in 23% (compared with 51% for younger patients), suggesting that age alone should be cautiously considered as a single decision-making factor [53].

Hypothermic cardiac arrest represents an important indication for eCPR, yet it still carries a high mortality rate primarily due to underlying primary asphyxia. In our study, only two patients with hypothermia survived. Asphyxia-related death by neurological criteria diagnosed within the first 3 days was the predominant cause of death. A previous meta-analysis involving 464 patients reported a considerably higher survival rate (37%) compared with our results, which could be attributed to the different aetiology of hypothermia, as the authors analysed a mixed population from 23 studies [57].

### Strengths and Limitations

This study has several limitations. Due to its retrospective nature, selection bias cannot be excluded. It is complex to discriminate ECMO-related adverse events from potential complications of an underlying disease and the direct cause of cardiac arrest. However, the reported initial rhythm and the duration of reanimation are related to the underlying disease, while the major bleeding could be a consequence of ECMO support and distorted coagulation. To identify and classify haemorrhage, we used the ELSO bleeding definition [13]; however, some adverse events may have been missed or overlooked if no additional diagnostic procedure was accomplished. Regarding patient outcomes, the GCS and CPC findings are limited for patients who were repatriated to home intensive care units, as they were still sedated or even on ECMO support. As follow-up was not possible, these patients were excluded from the CPC analysis, reducing the patient sample. However, repatriation was always initiated due to capacity or logistical reasons, often shortly after ECMO termination. Lastly, although our study includes a large cohort of patients, larger samples and further studies are needed to elucidate the role of eCPR in the survival of patients with cardiac arrest.

## 5. Conclusions

In this retrospective study from an ECMO referral centre, 46% of patients following eCPR survived until ICU discharge and 43% were discharged from the hospital, with the majority having favourable neurological outcomes. We identified asystole as an initial rhythm, longer duration of CPR, and CPR on the weekend as significant risk factors for ICU mortality. ECPR-related decision-making could be additionally supported by comprehension of the reported risk factors. Further studies are needed to elucidate the impact of peri-arrest variables on outcomes, aiming to improve patient selection for this invasive and resource-intensive procedure.

## Figures and Tables

**Table 1:** Demographic and clinical characteristics of included patients.

	All Patients (*n* = 90)	Survivors (*n* = 41)	Non-Survivors (*n* = 49)	*p*-Value	Missing Data (*n*/Total)
Age (years)	53.3 ± 17	54.2 ± 14	52.6 ± 19	0.656	0/90
0–30	8 (9)	2 (5)	6 (12)	0.067	0/90
31–60	53 (59)	29 (71)	24 (49)		
61–70	15 (17)	3 (7)	12 (25)		
>71	14 (16)	7 (17)	7 (14)		
Sex (male)	59 (66)	29 (71)	30 (61)	0.380	0/90
Height (m)	1.70 ± 0.17	1.73 ± 0.14	1.68 ± 0.20	0.153	11/90
Weight (kg)	77.8 ± 20.7	80.1 ± 20.6	75.6 ± 20.8	0.320	8/90
Body mass index (kg/m^2^)	25.9 ± 5.1	26.1 ± 5.0	25.7 ± 5.1	0.766	11/90
SOFA score	13 (7–17)	13 (7–16)	13 (11–17)	0.044	1/90
SAPS III score	80 (50–129)	76 (50–96)	83 (67–129)	<0.001	1/90
SAPS III score predicted mortality (%)	74 (17–98)	67 (17–89)	77 (50–98)	<0.001	1/90
**Cardiac arrest aetiology**					
Myocardial infarction	42 (47)	20 (49)	22 (45)	0.032	0/90
Hypothermia	14 (16)	2 (5)	12 (25)		
Heart intervention	10 (11)	5 (12)	5 (10)		
Cardiomyopathy	6 (7)	3 (7)	3 (6)		
Pulmonary embolism	3 (3)	0 (0)	3 (6)		
Other (cardiac)	6 (7)	5 (12)	1 (2)		
Other (primary non-cardiac) *	9 (10)	6 (15)	3 (6)		
**Location of cardiac arrest**					
Out-of-hospital	26 (29)	4 (10)	22 (45)	<0.001	0/90
In-hospital	58 (64)	32 (78)	26 (53)		
Retrieved from another centre on ECMO	6 (7)	5 (12)	1 (2)		
Witnessed arrest	75 (83)	41 (100)	34 (69)	<0.001	0/90
**Day of cardiac arrest**					
Weekday	65 (72)	35 (85)	30 (61)	0.017	0/90
Weekend	25 (28)	6 (15)	19 (39)		
**Initial rhythm**					
Shockable	40 (44)	23 (56)	17 (35)	0.056	0/90
Non-shockable	50 (56)	18 (44)	32 (65)		
**Initial rhythm (details)**					
Pulseless electrical activity	29 (32)	14 (34)	15 (31)	0.019	0/90
Asystole	21 (23)	4 (10)	17 (35)		
Ventricular fibrillation	35 (39)	19 (46)	16 (33)		
Pulseless ventricular tachycardia	5 (6)	4 (10)	1 (2)		
**CPR duration (minutes)**	45 (4–220)	32 (4–220)	60 (9–189)	0.004	3/90
<20	14 (16)	8 (21)	6 (13)	0.007	3/90
21–40	28 (32)	17 (44)	11 (23)		
41–60	18 (21)	9 (23)	9 (19)		
>60	27 (31)	5 (13)	22 (46)		
Peripheral cannulation	87 (97)	40 (98)	47 (96)	1.000	0/90
Percutaneous cannulation	71 (79)	35 (85)	36 (74)	0.202	0/90
ECMO duration (days)	3 (1–25)	5 (1–14)	2 (1–25)	0.001	0/90
ECMO support duration < 7 days	75 (83)	32 (78)	43 (88)	0.263	0/90
Length of ICU stay (days)	8 (1–121)	21 (2–121)	3 (1–56)	<0.001	0/90

Values are presented as the mean ± SD, median (minimum–maximum), or number (%) of patients. * Other (primary non-cardiac) includes intoxication (n = 1), intraoperative complications (n = 3), accidents (n = 2), and unknown causes (n = 3). ECMO: extracorporeal membrane oxygenation; SOFA: sequential organ failure assessment score; SAPS III: simplified acute physiology score III; ICU: intensive care unit; CPR: cardiopulmonary reanimation.

**Table 2:** Blood gas parameters (before and after ECMO initiation).

	All Patients (*n* = 90)	Survivors (*n* = 41)	Non-Survivors (*n* = 49)	*p*-Value	Missing Data (*n*/Total)
**Pre-ECMO blood gas parameters**					
Arterial pH	7.092 (6.400–7.484)	7.110 (6.789–7.385)	7.000 (6.400–7.484)	0.022	31/90
Venous pH	6.976 (6.500–7.376)	7.041 (6.800–7.238)	6.958 (6.500–7.376)	0.388	75/90
Lactate (mg/dL)	93 (8–235)	72 (10–155)	118 (8–235)	0.004	19/90
Potassium (mmol/L)	4.2 (2.7–10.7)	4.2 (2.7–9.1)	4.3 (2.7–10.7)	0.419	17/90
Glucose (mg/dL)	229 (64–537)	197 (64–486)	257 (70–537)	0.378	18/90
**Blood gas parameters after ECMO initiation**					
Arterial pH directly after ECMO initiation	7.040 (6.350–7.799)	7.123 (6.800–7.421)	6.972 (6.350–7.799)	<0.001	3/90
Lactate directly after ECMO initiation (mg/dL)	114 (14–265)	88 (14–207)	133 (35–265)	0.001	3/90
Arterial pH at 30 min	7.124 (6.600–7.418)	7.181 (6.907–7.352)	7.050 (6.600–7.418)	0.007	18/90
Lactate at 30 min (mg/dL)	119 (12–224)	89 (12–211)	130 (24–224)	<0.001	17/90
Potassium at 30 min (mmol/L)	3.7 (2.3–10.2)	3.6 (2.3–7.8)	3.8 (2.3–10.2)	0.776	17/90
Glucose at 30 min (mg/dL)	244 (49–476)	187 (49–476)	268 (59–438)	0.136	17/90

Values are presented as the mean ± SD and median (minimum–maximum). ECMO: extracorporeal membrane oxygenation.

**Table 3:** Patient outcomes and adverse events (during ICU stay and at hospital discharge).

	All Patients (*n* = 90)	Survivors (*n* = 41)	Non-Survivors (*n* = 49)	*p*-Value	Missing Data (*n*/Total)
**GCS at hospital discharge**	-	15 (3–15)	-	-	1/41
GCS 15	-	31 (78)	-	-	
GCS 14	-	1 (3)	-	-	
GCS 9	-	1 (3)	-	-	
GCS 3 (including sedation for repatriation to other ICU)	-	7 (18)	-	-	
**CPC at hospital discharge**					8/41
Persistent vegetative state	1 (1)	1 (3)	-	-	
Severe disability	2 (2)	2 (6)	-	-	
Moderate disability	3 (4)	3 (9)	-	-	
Good recovery	27 (33)	27 (82)	-	-	
**Adverse events**					
Haemorrhage	46 (51)	19 (46)	27 (55)	0.526	0/90
Major haemorrhage	25 (28)	5 (12)	20 (41)	0.004	0/90
Minor haemorrhage	21 (23)	14 (34)	7 (14)	0.044	0/90
Intracranial bleeding	9 (10)	2 (5)	7 (14)	0.173	0/90
Extremity ischemia	14 (16)	7 (17)	7 (14)	0.776	0/90
Compartment syndrome	8 (9)	2 (5)	6 (12)	0.283	0/90
Thrombosis	11 (12)	4 (10)	7 (14)	0.748	0/90
Acute kidney injury during ECMO support	52 (58)	24 (59)	28 (57)	1.000	0/90
Continuous renal replacement therapy during ECMO	41 (46)	20 (49)	21 (43)	0.672	0/90
**Reason for ECMO support termination**					
Improvement	42 (47)	33 (83)	9 (18)	<0.001	1/89
Death	37 (42)	0 (0)	37 (76)		
Bridge to other assistance (mechanical circulatory support) or heart transplantation	10 (11)	7 (18)	3 (6)		
Organ procurement	9 (10)	0 (0)	9 (18)	-	0/49
**Mortality**					
Death during ECMO	37 (41)	-	-	-	0/90
Death during ICU	49 (54)	-	-	-	0/90
Death during the same hospital stay	51 (57)	-	-	-	0/90
30-day mortality	48 (55)	-	-	-	3/90
60-day mortality	49 (64)	-	-	-	13/90
180-day mortality	51 (69)	-	-	-	16/90
One-year mortality	52 (71)	-	-	-	17/90

Values are presented as the median (minimum–maximum) and number (%) of patients. GCS: Glasgow coma scale; CPC: cerebral performance category score; ECMO: extracorporeal membrane oxygenation; ICU: intensive care unit; CPR: cardiopulmonary resuscitation.

**Table 4:** Identification of risk factors for death: multivariate analysis (n = 90).

Nondependent Variable	B-Coefficient	*p*-Value	HR	95% Confidence Interval	
				Lower	Upper
**Initial rhythm (reference category: ventricular fibrillation)**					
Asystole	1.057	0.049	2.88	1.01	8.25
Pulseless electrical activity	0.203	0.735	1.23	0.38	3.96
Pulseless ventricular tachycardia	0.236	0.835	1.27	0.14	11.7
**Location of cardiac arrest (reference category: in-hospital)**					
Out-of-hospital	0.080	0.943	1.08	0.12	9.59
Retrieved from another centre on ECMO	−13.728	0.976	0.00	-	-
**Cardiac arrest aetiology (reference category: myocardial infarction)**					
Heart intervention	0.031	0.963	1.03	0.28	3.87
Hypothermia	−0.756	0.425	0.47	0.07	3.02
Cardiomyopathy	−2.147	0.056	0.12	0.01	1.06
Other (cardiac)	−1.777	0.097	0.17	0.02	1.39
Other (primary non-cardiac)	−0.660	0.351	0.52	0.13	2.07
**Witnessed cardiac arrest**	−1.086	0.217	0.34	0.06	1.89
**CPR duration (minutes)**	0.015	0.043	1.02	1.00	1.03
**CPR on weekend**	0.942	0.032	2.57	1.08	6.08
**pH before ECMO initiation**	1.019	0.604	2.77	0.06	130.21
**Lactate before ECMO initiation (mg/dL)**	0.004	0.425	1.00	0.99	1.02

Blood gas parameters after ECMO initiation were excluded from the model due to potential collinearity. Abbreviations: CPR: cardiopulmonary resuscitation; eCPR: extracorporeal cardiopulmonary resuscitation ECMO: extracorporeal membrane oxygenation; HR: hazard ratio.

## Data Availability

The data presented in this study are available upon reasonable request from the corresponding author.

## Supplementary Materials

The following supporting information can be downloaded at: https://www.mdpi.com/article/10.3390/jcdd11090272/s1, Table S1. STROBE Statement—checklist of items that should be included in reports of cohort studies; Table S2. Serial measurements of arterial blood gas; Figure S1. Kaplan–Meier mean estimate of overall ICU mortality in regard to initial rhythm (n = 90); Table S3. Univariate analyses: Identification of risk factors and predictors for ICU mortality (n = 90).
