# Supplementary material for: Mortality Predictors and Neurological Outcomes Following Extracorporeal Cardiopulmonary Resuscitation (eCPR): A Single-Center Retrospective Study
Source: J Cardiovasc Dev Dis. 2024 Sep 2;11(9):272. doi: 10.3390/jcdd11090272 (PMC11432051; doi:10.3390/jcdd11090272)
Supplement: Supplementary file 1 [file jcdd-11-00272-s001.zip › jcdd-3172075-supplementary.pdf]

**Supplementary material**

# Mortality Predictors and Neurological Outcomes Following Extracorporeal Cardiopulmonary Resuscitation (eCPR): A Single-Center Retrospective Study

## Contents

**Table S1.** STROBE Statement - Checklist of items that should be included in reports of cohort studies

**Table S2.** Serial measurements of arterial blood gas

**Figure S1.** Kaplan-Meier mean estimate of overall ICU mortality in regard of initial rhythm (n = 90)

**Table S3.** Univariate analyses: Identification of risk factors and predictors for ICU-mortality (n = 90)

**Table S1.** STROBE Statement - Checklist of items that should be included in reports of cohort studies

| No. | Item | Recommendation | Page |
|---|---|---|---|
| | **Title and abstract** | | |
| 1 | | (*a*) Indicate the study's design with a commonly used term in the title or the abstract | 1 |
| | | (*b*) Provide in the abstract an informative and balanced summary of what was done and what was found | 1 |
| | **Introduction** | | |
| 2 | Background/rationale | Explain the scientific background and rationale for the investigation being reported | 1-2 |
| 3 | Objectives | State specific objectives, including any prespecified hypotheses | 1-2 |
| | **Methods** | | |
| 4 | Study design | Present key elements of study design early in the paper | 2-3 |
| 5 | Setting | Describe the setting, locations, and relevant dates, including periods of recruitment, exposure, follow-up, and data collection | 2-3 |
| 6 | Participants | (*a*) Give the eligibility criteria, and the sources and methods of selection of participants. Describe methods of follow-up | 2-3 |
| | | (*b*) For matched studies, give matching criteria and number of exposed and unexposed | 2-3 |
| 7 | Variables | Clearly define all outcomes, exposures, predictors, potential confounders, and effect modifiers. Give diagnostic criteria, if applicable | 2-3 |
| 8* | Data sources/ measurement | For each variable of interest, give sources of data and details of methods of assessment (measurement). Describe comparability of assessment methods if there is more than one group | 2-3 |
| 9 | Bias | Describe any efforts to address potential sources of bias | 2-3 |
| 10 | Study size | Explain how the study size was arrived at | NA |
| 11 | Quantitative variables | Explain how quantitative variables were handled in the analyses. If applicable, describe which groupings were chosen and why | 2-3 |
| 12 | Statistical methods | (*a*) Describe all statistical methods, including those used to control for confounding | 2-3 |
| | | (*b*) Describe any methods used to examine subgroups and interactions | 2-3 |
| | | (*c*) Explain how missing data were addressed | 2-3 |
| | | (*d*) If applicable, explain how loss to follow-up was addressed | NA |
| | | (*e*) Describe any sensitivity analyses | NA |
| | **Results** | | |
| 13* | Participants | (a) Report numbers of individuals at each stage of study—eg numbers potentially eligible, examined for eligibility, confirmed eligible, included in the study, completing follow-up, and analysed | 3-8 |
| | | (b) Give reasons for non-participation at each stage | 3-4 |
| | | (c) Consider use of a flow diagram | NA |
| 14* | Descriptive data | (a) Give characteristics of study participants (eg demographic, clinical, social) and information on exposures and potential confounders | Table 1 |
| | | (b) Indicate number of participants with missing data for each variable of interest | Tables |
| | | (c) Summarise follow-up time (eg, average and total amount) | Tables |
| 15* | Outcome data | Report numbers of outcome events or summary measures over time | Tables |
| 16 | Main results | (*a*) Give unadjusted estimates and, if applicable, confounder-adjusted estimates and their precision (eg, 95% confidence interval). Make clear which confounders were adjusted for and why they were included | Tables |
| | | (*b*) Report category boundaries when continuous variables were categorized | Tables |
| | | (*c*) If relevant, consider translating estimates of relative risk into absolute risk for a meaningful time period | NA |
| 17 | Other analyses | Report other analyses done—eg analyses of subgroups and interactions, and sensitivity analyses | 3-8 |
| | **Discussion** | | |
| 18 | Key results | Summarise key results with reference to study objectives | 8-10 |
| 19 | Limitations | Discuss limitations of the study, taking into account sources of potential bias or imprecision. Discuss both direction and magnitude of any potential bias | 10-11 |
| 20 | Interpretation | Give a cautious overall interpretation of results considering objectives, limitations, multiplicity of analyses, results from similar studies, and other relevant evidence | 10-11 |
| 21 | Generalisability | Discuss the generalisability (external validity) of the study results | 10-11 |
| | **Other information** | | |
| 22 | Funding | Give the source of funding and the role of the funders for the present study and, if applicable, for the original study on which the present article is based | 10-11 |

*Give information separately for exposed and unexposed groups.

**Table S2.** Serial measurements of arterial blood gas (after ECMO initiation)

| | All patients (n = 90) | Survivors (n = 41) | Non-survivors (n = 49) | p-value | Missing data (n/total) |
|---|---|---|---|---|---|
| Serial arterial pH measurements | | | | | |
| 1h | 7.230 (6.700-7.428) | 7.261 (6.923-7.428) | 7.200 (6.700-7.420) | 0.056 | 23/90 |
| 2h | 7.277 (6.800-7.588) | 7.308 (7.054-7.588) | 7.249 (6.800-7.509) | 0.007 | 12/90 |
| 6h | 7.362 (7.121-7.597) | 7.401 (7.172-7.597) | 7.323 (7.121-7.487) | 0.001 | 13/90 |
| 12h | 7.401 (7.161-7.596) | 7.402 (7.199-7.596) | 7.380 (7.161-7.530) | 0.235 | 19/90 |
| 18h | 7.401 (7.081-7.638) | 7.410 (7.270-7.638) | 7.381 (7.081-7.540) | 0.134 | 24/90 |
| 24h | 7.393 (7.195-7.542) | 7.404 (7.203-7.517) | 7.370 (7.195-7.542) | 0.174 | 26/90 |
| 48h | 7.429 (7.255-7.611) | 7.425 (7.255-7.611) | 7.451 (7.287-7.589) | 0.828 | 40/90 |
| Serial lactate levels measurements | | | | | |
| 1h | 104 (9-214) | 85 (9-192) | 112 (22-214) | 0.001 | 24/90 |
| 2h | 94 (7-228) | 65 (7-163) | 112 (19-228) | <0.001 | 11/90 |
| 6h | 60 (8-197) | 37 (8-116) | 77 (16-197) | <0.001 | 13/90 |
| 12h | 38 (3-179) | 26 (3-109) | 54 (12-179) | 0.003 | 19/90 |
| 18h | 23 (5-139) | 19 (5-90) | 41 (10-139) | <0.001 | 24/90 |
| 24h | 19 (5-119) | 15 (5-51) | 27 (10-119) | <0.001 | 26/90 |
| 48h | 17 (4-65) | 13 (4-32) | 23 (11-65) | 0.002 | 40/90 |

Values are presented as mean ± SD and median (minimum - maximum). ECMO: extracorporeal membrane oxygenation.

**Figure S1.** Kaplan-Meier mean estimate of overall ICU mortality in regard of initial rhythm: 25 days for non-shockable (n = 50; 95% CI 17.9-32.9), and 38 days for shockable rhythm (n = 40; 95% CI 29.4-46.2), p = 0.044. Abbreviations: eCPR, extracorporeal cardiopulmonary reanimation.

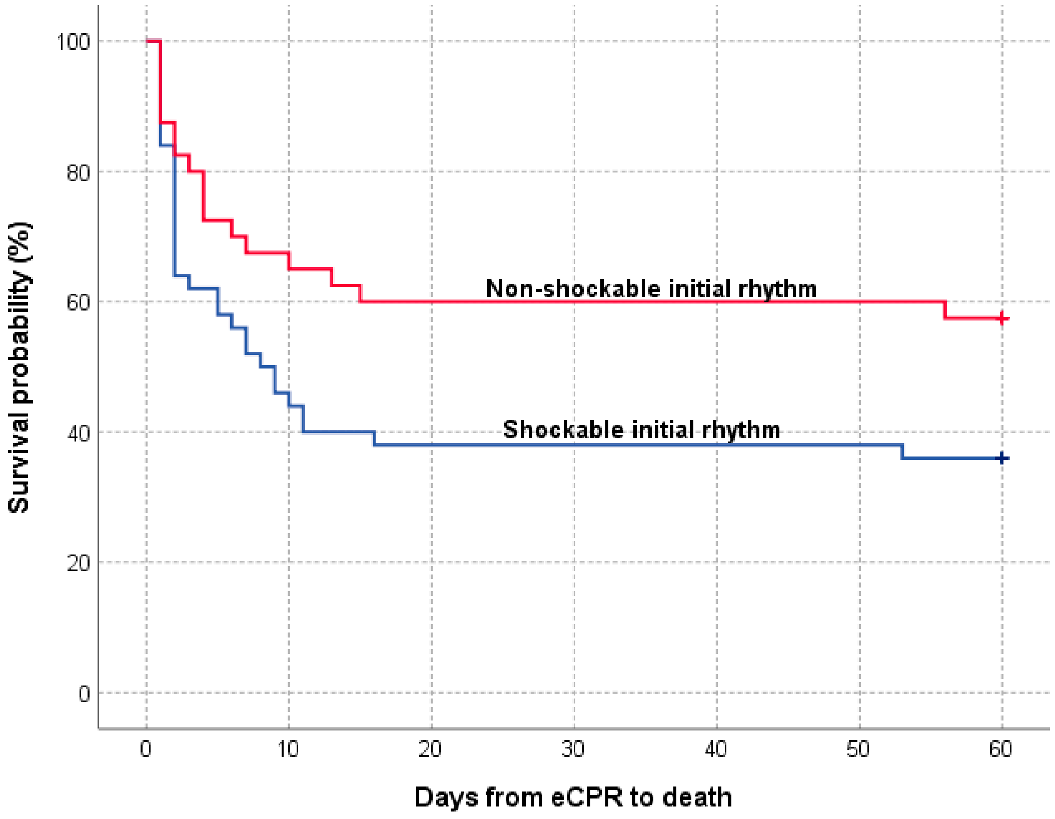

**Table S3.** Univariate analyses: Identification of risk factors and predictors for ICU-mortality (n = 90)

| Nondependent variable | B-coefficient | P value | HR | 95% confidence interval lower | upper |
|---|---|---|---|---|---|
| Age (years) | -0.004 | 0.612 | 1.00 | 0.98 | 1.01 |
| Sex (male/female) | 0.223 | 0.447 | 1.05 | 0.70 | 2.22 |
| BMI (kg/m$^2$) | -0.013 | 0.680 | 0.99 | 0.93 | 1.05 |
| Witnessed cardiac arrest | -1.287 | <0.001 | 0.28 | 0.15 | 0.52 |
| CPR duration (minutes) | 0.007 | 0.005 | 1.01 | 1.00 | 1.01 |
| Peripheral ECMO cannulation | 0.531 | 0.463 | 1.70 | 0.41 | 7.02 |
| Day of cardiac arrest (weekend) | 0.726 | 0.014 | 2.07 | 1.16 | 3.68 |
| Cardiac arrest aetiology (reference category: Myocardial infarction) | | | | | |
| Heart valve intervention | -0.155 | 0.755 | 0.86 | 0.32 | 2.26 |
| Hypothermia | 0.769 | 0.033 | 2.16 | 1.06 | 4.38 |
| Cardiomyopathy | -0.058 | 0.925 | 0.94 | 0.28 | 3.16 |
| Other (cardiac aetiology) | -1.387 | 0.175 | 0.25 | 0.03 | 1.85 |
| Pulmonary embolism | 0.783 | 0.206 | 2.19 | 0.65 | 7.36 |
| Other primary non-cardiac | -0.656 | 0.287 | 0.52 | 0.16 | 1.74 |
| Initial rhythm | | | | | |
| Initial rhythm (non-shockable initial rhythm) | -0.575 | 0.056 | 0.56 | 0.31 | 1.02 |
| Initial rhythm (reference category: ventricular fibrillation) | | | | | |
| Pulseless electrical activity | 0.913 | 0.009 | 2.49 | 1.25 | 4.96 |
| Asystole | 0.131 | 0.716 | 1.14 | 0.56 | 2.31 |
| Pulseless ventricular tachycardia | -1.092 | 0.290 | 0.34 | 0.04 | 2.53 |
| Location of cardiac arrest (reference category: In-hospital) | | | | | |
| Out-of-hospital | 1.036 | <0.001 | 2.82 | 1.58 | 5.02 |
| Retrieved from another centre on ECMO | -1.175 | 0.249 | 0.31 | 0.04 | 2.28 |
| Complications | | | | | |
| Haemorrhage | 0.195 | 0.496 | 1.22 | 0.69 | 2.14 |
| Major Haemorrhage | 0.411 | 0.005 | 1.51 | 1.13 | 2.01 |
| Minor Haemorrhage | -0.788 | 0.054 | 0.46 | 0.20 | 1.01 |
| Intracranial bleeding | 0.416 | 0.309 | 1.52 | 0.68 | 3.38 |
| Extremity ischemia | -0.182 | 0.656 | 0.83 | 0.37 | 1.86 |
| Compartment syndrome | 0.539 | 0.217 | 1.71 | 0.73 | 4.04 |
| Thrombosis | 0.094 | 0.819 | 1.10 | 0.49 | 2.45 |
| Acute kidney injury during ECMO support | -0.173 | 0.549 | 0.84 | 0.48 | 1.48 |
| Continuous renal replacement therapy during ECMO | -0.348 | 0.229 | 0.71 | 0.40 | 1.25 |
| Blood gas parameters before ECMO initiation | | | | | |
| Arterial pH before ECMO | -1.993 | 0.006 | 0.14 | 0.03 | 0.56 |
| Venous pH before ECMO | -0.869 | 0.637 | 0.42 | 0.01 | 15.56 |
| Lactate (mg/dL) before ECMO | 0.008 | 0.003 | 1.01 | 1.00 | 1.01 |
| Potassium (mmol/L) before ECMO | 0.105 | 0.243 | 1.11 | 0.93 | 1.32 |
| Glucose (mg/dL) | 0.002 | 0.194 | 1.00 | 1.00 | 1.00 |
| Blood gas parameters after ECMO initiation | | | | | |
| Arterial pH directly after ECMO initiation | -2.798 | <0.001 | 0.06 | 0.02 | 0.25 |
| Lactate directly after ECMO initiation (mg/dL) | 0.012 | <0.001 | 1.01 | 1.01 | 1.02 |
| Arterial pH at 30min | -2.778 | 0.001 | 0.06 | 0.01 | 0.30 |
| Lactate at 30min (mg/dL) | 0.012 | <0.001 | 1.01 | 1.01 | 1.02 |
| Potassium at 30min (mmol/L) | 0.015 | 0.901 | 1.02 | 0.80 | 1.29 |
| Glucose at 30min (mg/dL) | 0.002 | 0.149 | 1.00 | 1.00 | 1.01 |

ICU: intensive care unit; ECMO: extracorporeal membrane oxygenation; CPR: cardiopulmonary resuscitation
